# Supporting adolescents’ mental health during COVID-19 by utilising lessons from the aftermath of the Great East Japan Earthquake

**DOI:** 10.1057/s41599-022-01330-1

**Published:** 2022-09-23

**Authors:** Junko Okuyama, Shin-Ichi Izumi, Shunichi Funakoshi, Shuji Seto, Hiroyuki Sasaki, Kiyoshi Ito, Fumihiko Imamura, Mayumi Willgerodt, Yu Fukuda

**Affiliations:** 1 Designated National University, Core Research Cluster of Disaster Science, Tohoku University, Sendai, Japan; 2 Department of Rehabilitation, Tohoku University, Sendai, Japan; 3 Miyagi Psychtric Center, Natori, Japan; 4 International Research Institute of Disaster Science, Tohoku University, Sendai, Japan; 5 Department of Child, Family, and Population Health Nursing at the University of Washington, Seattle, USA; 6 Notre Dame Seishin University, Okayama, Japan

**Keywords:** Health humanities, Psychology

## Abstract

Historical data can determine how adolescents recover from difficult situations such as the Coronavirus disease 2019 (COVID-19) pandemic. This study analysed 3 years of data obtained from high-school students who had been affected by the 2011 Great East Japan Earthquake and consequently evidenced the importance of increasing resilience among affected adolescents. This involved identifying factors contributing to resilience through a model that assessed for each tsunami disaster. This model was determined by assessing the correlation between survivors’ resilience scores and their measured psychological and lifestyle scores. This approach showed that, in all tsunami damage models, resilience was most affected by the depressed emotions. Thus, our approach suggests that interventions for improving the depressed mood may improve resilience in adolescents during the COVID-19 pandemic.

## Introduction

Japan is experiencing increasingly high rates of suicide among adolescents. It is the only developed country where suicide is the leading cause of death among those aged 15−39 years, with a particularly high incidence of adolescent suicide (Okada et al., 2022). The current prolonged COVID-19 pandemic may be even more stressful for Japanese high-school students, who were already stressed out to begin with (Okuyama and Seto, 2021).

Not only in Japan but globally, recent research has shown that many adolescents are feeling pressured to control the transmission of the SARS-CoV-2 virus, which causes COVID-19 (de Figueiredo et al., 2020). Stressful life events, prolonged home confinement, the cruelty of grief, family violence, and abuse of the Internet and social media can significantly affect adolescents’ mental health (Afrin et al., 2022). The COVID-19 pandemic and attendant lockdowns have been associated with post-traumatic stress, depression disorders, anxiety disorders, and other psychiatric disorders and may increase the incidence of grief-related symptoms.

The field of disaster psychiatry aims to understand the psychological, biological, social, and environmental factors affecting survivors of disaster or traumatic situations and develop interventions and treatments for restoring and maintaining their mental health. Common estimates show that 10% of children and adolescents have significant mental health problems affecting their day-to-day lives, which, if left untreated, could continue into their adulthoods (Membride, 2016). During disasters, adolescents experience an increase in mental health issues (Ahmed et al., 2020; Ollendick and Hoffmann 1982; Pfledderer et al., 2019; Xiang et al., 2020).

In Japan, disaster psychiatry research has been ongoing since the Great East Japan Earthquake of March 11, 2011, and it has produced some very significant findings that can be applied towards addressing the COVID-19 pandemic’s impact on adolescents’ mental health and social functioning. However, thus far, no research data have suggested any ways to avoid this COVID-19-induced mental health crisis (Guessoum et al., 2020; Pfefferbaum and North, 2020).

The findings of disaster psychiatry so far can be applied towards supporting and increasing adolescents’ resilience during the COVID-19 pandemic. This study aims to provide some effective support for increasing adolescent resilience during the COVID-19 pandemic by using data from the aftermath of the Great East Japan Earthquake. We modelled, evaluated, and analysed factors associated with the Great East Japan Earthquake’s effect on adolescents’ resilience. This is necessary for promoting adolescents’ healthy growth under the current pandemic conditions and for reducing the risk of this age group developing such mental illnesses.

## Similarities between the COVID-19 pandemic conditions and the 2011 Great East Japan Earthquake

**Fig. 1:**
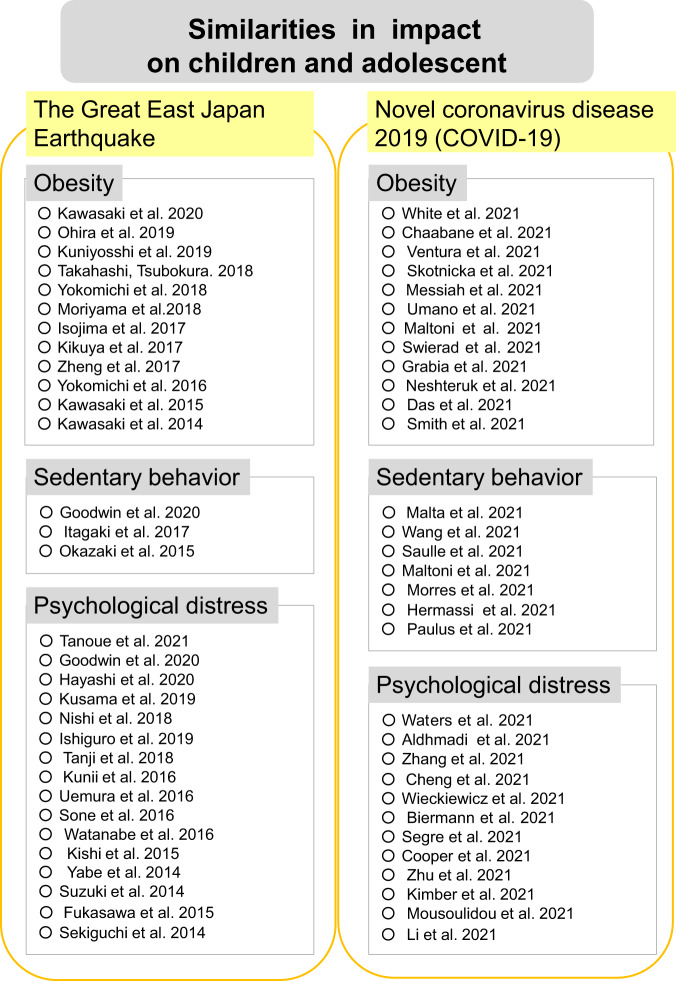
Adolescent homology during the COVID-19 pandemic and after the Great East Japan earthquake. On the right “The Great East Japan Earthquake” gives the articles on children and adolescents after the Great East Japan Earthquake, and on the left “Novel coronavirus disease 2019 (COVID-19)” gives the articles on children andadolescents under the COVID-19 pandemic.

In Japan, schools closed temporarily, and people refrained from going out in order to prevent the spread of the COVID-19 infection. The Japanese Ministry of Education, Culture, Sports, Science and Technology (MEXT) decided to close schools nationwide from February 28, 2020 until before spring break; furthermore, many schools remained closed until May 31, 2020. Figure 1 summarises previous studies’ findings on the impact of the COVID-19 pandemic on adolescents. The major categories of this impact are (1) obesity, (2) sedentary behaviour, and (3) psychological distress. Among children and adolescents after the Great East Japan Earthquake, there are the previous studies by Kawasaki et al. (2020), Ohira et al. (2019), Kuniyoshi et al. (2019), Takahashi and Tsubokura (2018), Yokomichi et al. (2018), Moriyama et al. (2018), Isojima et al. (2017), Kikuya et al. (2017), Zheng et al. (2017), Yokomichi et al. (2016), Kawasaki et al. (2015) and Kawasaki et al. (2014) on obesity. And there are previous studies on the sedentary behavior by Goodwin et al. (2020), Itagaki et al. (2017) and Okazaki et al (2015). For the psychological distress, there are the previous studies by Tanoue et al. (2021), Goodwin et al. (2020), Hayashi et al. (2020), Kusama et al. (2019), Nishi et al. (2018), Ishiguro et al. (2019), Tanji et al. (2018), Kunii et al. (2016), Uemura et al. (2016), Sone et al. (2016), Watanabe et al. (2016), Kishi et al. (2015), Yabe et al. (2014), Suzuki et al. (2014), Fukasawa et al. (2015) and Sekiguchi et al. (2014). And under COVID-19 pandemic, there are the previous studies by White et al. (2021), Chaabane et al. (2021), Ventura et al. (2021), Skotnicka et al. (2021), Messiah et al. (2021), Umano et al. (2021), Maltoni et al. (2021), Swierad et al. (2021), Grabia et al. (2021), Neshteruk et al. (2021), Das et al. (2021) and Smith et al. (2021). And there are previous studies on the sedentary behavior by Malta et al. (2021), Wang et al. (2021), Saulle et al. (2021), Maltoni et al. (2021), Morres et al. (2021), Hermassi et al. (2021) and Paulus et al. (2021). For the psychological distress, there are the previous studies by Waters et al. (2021), Aldhmadi et al. (2021), Zhang et al. (2021), Cheng et al. (2021), Wieckiewicz et al. (2021), Biermann et al. (2021), Segre et al. (2021), Cooper et al. (2021), Zhu et al. (2021), Kimber et al. (2021), Mousoulidou et al. (2021) and Li et al. (2021).

It was found that adolescents affected by the Great East Japan Earthquake experience similar impacts to those of adolescents currently affected by the COVID-19 pandemic (Fig. 1). Japanese youth affected by the Great East Japan Earthquake in 2011 could not experience their farewell party, which was usually held in March, or their entrance ceremony, which was usually held in April. Interactions among peers, such as club activities, were also cancelled, and this reduced adolescents’ motivation (Kitami and Nakashita, 2017). In addition to Japanese high schoolers’ events, we also focused on children and adolescents’ lives after the Great East Japan Earthquake and found that they were homologous to youngsters’ daily lives during the COVID-19 pandemic (Okuyama et al., 2021a). Specifically, teaching methods changed, after-school activities such as club activities disappeared, and youngsters did not leave their homes to meet with their peers or travel far away from home for leisure activities.

School is not only an educational hub, but also a home outside the home with plentiful free space. Schools offer windows of freedom, scope of interaction with fellows and seniors, and psychological solace, apart from providing pedagogy and scholastics. Schools play an edifying role in promoting the importance of personal hygiene, physical activity, and healthy food and bodily habits (Ghosh et al., 2020; Sylva, 1994).

In the aftermath of the earthquake, many victims were forced to change their personal habits (e.g., lifestyle and exercise patterns); this development is like the conditions faced by the public throughout the COVID-19 pandemic (Hayashi and Tomita, 2012). Consequently, we hypothesised that longitudinal data on adolescents’ lives after the Great East Japan Earthquake would help us devise future interventions for predicting and improving the psychological states of the youngsters who are currently being affected by the COVID-19 pandemic and lockdown. In particular, children and adolescents affected by the Great East Japan Earthquake reported that their physical activity levels decreased (Okazaki et al., 2015). In more recent times, one study has indicated that Latin American adolescents have experienced a pronounced decrease in physical activity during the COVID-19 pandemic (Ruíz-Roso et al., 2020).

## Methods

### Participants

**Fig. 2:**
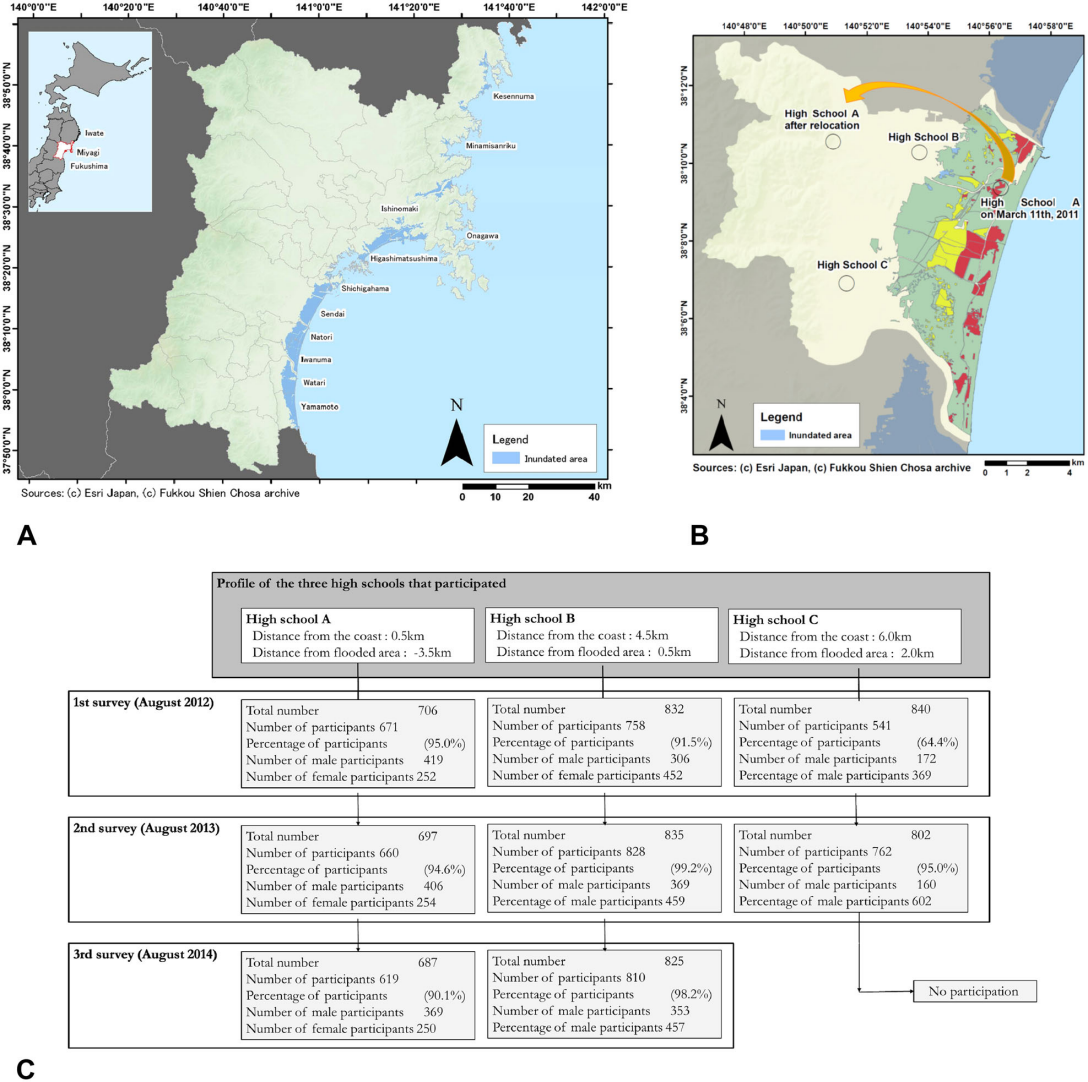
Longitudinal study of high-school students in the 3 years following the Great East Japan Earthquake. **A** Flooded areas in Miyagi Prefecture by the Great East Japan Earthquake tsunami. **B** Flooded areas in Miyagi Prefecture by the Great East Japan Earthquake tsunami. **C** Survey timing and number of participants from the three high schools.

The study participants were recruited from three high schools in the southern part of the Miyagi prefecture, which was hit by the 2011 Great East Japan Earthquake and the tsunami (Fig. 2A; Funakoshi et al., 2014). Most of the high-school students who experienced the earthquake in the Miyagi prefecture (approximate seismic intensity: 6) shared disaster-related experiences and repeatedly witnessed devastation in their residential areas regardless of their high school or grade level. High Schools A and B are in the city of Natori in the Yuriage area on the ocean side of the Miyagi prefecture. The Yuriage area was severely damaged by the tsunami and suffered an unprecedented 753 deaths (City of Natori, 2012). High School C is located inland in Iwanuma near Natori, unlike the other two schools (Ono et al., 2013; Funakoshi et al., 2014) (Fig. 2A).

These three high schools participated in this study across a period of 3 years—that is, from August 2012–August 2014.

In High School A, 671 students (95.0% of the total) participated in the first year, 758 (91.5%) in the second year, and 671 (95.0%) in the third year. In High School B, 660 (94.6%) participated in the first year, 660 (99.2%) in the second year, and 762 (95.0%) in the third year. In High School C, 619 (90.1%) participated in the first year, and 810 (98.2%) participated in the second year but not in the third year (Funakoshi et al., 2014; Okuyama et al., 2017a; Okuyama et al., 2017b; Okuyama et al., 2018) (Fig. 2C).

### Instruments

We used the Quick Inventory of Depressive Symptomatology Japanese version (QIDS-J), Self-Rating Anxiety Scale (SAS), the Impact of Event Scale—Revised (IES-R), and the 10-item Connor–Davidson Resilience Scale (CD-RISC-10).

#### Depressive symptoms

The Quick Inventory of Depressive Symptomatology Japanese version (QIDS-J) (Rush et al., 2003; Fijisawa et al., 2010), containing 16 questions, was used to measure respondents’ symptoms of depression. The reliability and validity of the Japanese version, the QIDS-J, have been confirmed (Fujikawa et al., 2010). The QIDS-J is a 16-item checklist. Scores greater than the cut-off point of 11 indicate moderate or severe levels of a depressive state.

#### Anxiety

The SAS is a measure developed by Zung et al. (1990), in which respondents are required to answer 20 questions regarding experiencing symptoms of anxiety (Olatunji et al., 2006). SAS scores over the cut-off point of 40 indicate a state of anxiety. The Japanese version of the SAS, which was used in the present study, had satisfactory internal consistency (Okamura et al., 1991); however, no relevant related data are available for Japanese adolescents. The mean SAS score for Chinese medical students (aged 15–21 years) is 47.60 (Tempski et al., 2015).

#### Post-traumatic stress reaction

The Impact of Event Scale—Revised (IES-R) (Horowitz et al., 1979) contains 22 questions on experiencing symptoms of earthquake-related post-traumatic stress reactions (PTSR), that respondents are required to answer. A higher total score indicates a greater psychological impact from the relevant event on the respondent (Crabbe et al., 2004). Participants with IES-R scores greater than 25 are considered to exhibit a significant PTSR (Asukai et al., 2002).

#### 10-item Connor–Davidson Resilience Scale

The 10-item Connor–Davidson Resilience Scale (CD-RISC-10) (Den et al., 2008; Saito and Okayasu, 2009; Connor and Davidson, 2003) focuses on 10 questions regarding the respondents’ experience of resilience after the earthquake. Each item is rated on a 5-point scale from 0 (not true at all) to 4 (true nearly all the time). Thus, the total score can vary from 0 to 40. Ye et al. (2017) have proposed that a CD-RISC-10 cut-off score below 25.5 should be considered as a reason for concern.

*Attitudinal data* regarding gender, survey ID, and age were collected.

*Questions about daily life* included self-reports of waking and sleeping times, time spent playing outside, watching TV, playing games, and whether breakfast was consumed.

Details of contributing participants are presented in Fig. 2B.

### Procedure

With the cooperation of the X Board of Education, we first explained the purpose and methods of the survey to three high schools in the southern part of the prefecture, and then obtained their consent to cooperate in the survey. After receiving consent, we sent an explanatory letter (a document explaining the purpose and methods of the survey and requesting cooperation) and a survey questionnaire to the schools and asked them to distribute them to students and their parents. Only those schools that gave their consent to obtain information about the students were asked to fill out the questionnaire about the students’ situation. In conducting this survey, we gave due consideration to the privacy and human rights of the students and explained the following to the students and their parents/guardians: (1) Only the student’s student ID number would be written on the survey form, and privacy would be strictly protected by thorough management of personal information; (2) Cooperation in the survey will be decided freely by the student and their guardian; (3) If the student does not wish to cooperate, they may not fill out and submit the form; (4) If the student does not cooperate in the survey, they will not be disadvantaged; and (5) The research results obtained from the survey may be published in academic conferences or journals, but will not be used for any other purpose. We asked the respondents to fill out and submit the survey form only if they agreed to participate in the survey. Only the ID number was entered in the survey form. The person analysing the survey results used an anonymized ID to analyse the information so that individuals could not be identified. The survey forms collected from each school were converted to electronic data by Hokkaido University and analysed by Miyagi Psychiatric Centre and Tohoku University. The study design, interpretation of the results in a manner that did not concern primary data, and data analysis policies were discussed among Miyagi Psychiatric Centre, Tohoku University, and University of Washington.

For all three high schools, the first survey was conducted in July 2012 (16 months after the earthquake), the second in July 2013 (28 months after the earthquake), and the third in July 2014 (40 months after the earthquake; Fig. 2B). These timings were set to coincide with the conclusion of the Miyagi Prefecture High School Comprehensive Athletic Meet and before the start of summer vacation in almost all Japanese high schools.

### Data analyses

The participants were in the first through third grades of a typical Japanese high school, and were between the ages of 15 and 18.

The statistical analysis was performed using IBM SPSS for Windows 24 (IBM Corp., Armonk, NY, USA) using a significance level of *P* < 0.05. Non-normally distributed data were expressed as medians (25th–75th percentile) for variables with a skewed distribution. The Mann–Whitney *U*-test was used to compare two groups of independent variables. Kruskal–Wallis test was performed to compare multiple independent variables. When applicable, post hoc adjusted comparisons with the Mann–Whitney *U*-test with Bonferroni correction were applied.

The contribution of psychological state and daily life to the factor resilience score was assessed using Prediction One (Sony Network Communications Inc. https://predictionone.sony.biz/). Prediction One software uses “Neural Network Libraries” (https://nnabla.org/), which is an open‐source software for deep learning. Based on the learning items, it automatically performs machine‐learning analysis, such as neural networks and gradient boosting trees, and easily and rapidly creates a prediction model by cross‐validation. The prediction model calculates the predicted value by entering the items of each patient. Despite no information on the details of its analysis, this software, which had been created originally for nonmedical applications, can be applied in various medical fields (Katsuki et al., 2020). The proposed model was estimated independently for each high school due to the varying effects of the tsunami on the confounding and response variables.

## Results

### Longitudinal changes in resilience and mental health indicators

Regarding CD-RISC-10 scores, in the 3-year observation of High School A students, the Kruskal–Wallis test showed a significant difference (*P* = 0.001; Table 1). A Bonferroni post hoc correction for multiple testing indicated that the 2014 CD-RISC-10 scores were significantly higher than those from 2012 and 2013 (*P* < 0.05). For the 3-year observation of High School B students, the Kruskal–Wallis test showed a significant difference (*P* = 0.021; Table 1). A Bonferroni post hoc correction for multiple testing indicated that the 2014 CD-RISC-10 scores were significantly higher than those for 2012 (*P* < 0.05). In the 2-year observation of High School C students, the Mann–Whitney-*U*-test showed that the 2012 CD-RISC-10 scores were significantly higher than those for 2013 (*P* = 0.000; Table 1).

**Table 1:** Resilience and mental health indicators of students assessed from 2012 to 2014.

Year of survey		High School A	(*n*)	High School B	(*n*)	High School C	(*n*)
*QIDS-J*							
Total		5 [3–9]	1810	5 [2–8]	2277	4 [2–7]	1140
2012		6 [3–10]	603	5 [3–9]	696	5 [2–8]	491
2013		5 [3–9]	614	4 [2–8]	770	4 [2–7]^c^	649
2014		5 [2–8]^a^	593	4 [2–7]^a^	811		
Kruskal–Wallis test	*χ*^2^	17.726		35.983			
	df	2		2			
	*P*	<0.001		<0.001			
Mann–Whitney test	U					141,671.50	
	Z					−3.221	
	*P*					0.001	
*SAS*							
Total		20 [15–26]	1706	22 [17–28]	2391	41 [37–44]	999
2012		40 [36–44]	598	42 [38–45]	690	41 [37–44]	438
2013		40 [36–43]	570	41 [39–44]	764	41 [37–44]	561
2014		40 [37–43]	538	41 [38–44]^b^	733		
Kruskal–Wallis test	*χ*^2^	0.572		6.786			
	df	2		2			
	*P*	0.751		0.034			
Mann–Whitney test	*U*					120,103.00	
	*Z*					−0.610	
	*P*					0.542	
*IES-R*							
Total		7 [1–22]	1949	4 [0–13]	2401	5 [0–19]	1303
2012		9 [2–24]	671	6 [1–16]	761	7 [1–20]	541
2013		7 [1–22]	660	4 [0–11]^c^	828	4 [0–16.25]^c^	762
2014		5 [0–19]^a^	618	3 [0–13]^a^	812		
Kruskal–Wallis test	*χ*^2^	19.406		23.118			
	df	2		2			
	*P*	<0.001		<0.001			
Mann–Whitney test	*U*					178,432.00	
	*Z*					−4.198	
	*P*					<0.001	
*CD-RISC-10*							
Total		20 [14–25]	1945	22 [17–28]	2400	20 [14–25]	1274
2012		19 [13–25]	667	21 [16–27]	760	20 [16–26]	513
2013		20 [13–25]	660	22 [17–29]	828	20 [10.5–25]^c^	761
2014		20 [16–26]^a,b^	218	20 [18–28]^a^	812		
Kruskal–Wallis test	*χ*^2^	14.126		7.768			
	df	2		2			
	*P*	<0.001		0.021			
Mann–Whitney test	*U*					167,368.00	
	*Z*					−4.333	
	*P*					<0.001	

Numerical variables with skewed distributions are expressed as the median [25th–75th percentile].

*QIDS-J* Quick Inventory of Depressive Symptomatology—Japanese version, *SAS* Zung Self-Rating Anxiety Scale, *IES-R* impact of event scale—revised, *CD-RISC-10* 10-item Conner-Davidson Resilience Scale.

^a^According to the post hoc Bonferroni test, there were differences in the scores between 2012 and 2014 (*P* < 0.05).

^b^According to the post hoc Bonferroni test, there were differences in the scores between 2013 and 2014 (*P* < 0.05).

^c^According to the post hoc Bonferroni test, there were differences in the scores between 2012 and 2013 (*P* < 0.05).

Regarding QIDS-J scores, in the 3-year observation of High School A students, the Kruskal–Wallis test showed a significant difference (*P* = 0.000; Table 1). A Bonferroni post hoc correction for multiple testing indicated that the 2012 QIDS-J scores were significantly higher than those for 2014 (*P* < 0.05). For the 3-year observation of High School B students, the Kruskal–Wallis test showed a significant difference (*P* = 0.000; Table 1). A Bonferroni post hoc correction for multiple testing indicated that the 2012 QIDS-J scores were significantly higher than the 2014 scores (*P* < 0.05). In the 2-year observation of High School C students, the Mann–Whitney-*U*-test showed that the 2012 QIDS-J scores were significantly higher than those for 2013 (*P* = 0.000; Table 1).

Regarding SAS scores, in the 3-year observation of High School A students, the Kruskal–Wallis test showed no significant difference (*P* = 0.751; Table 1). For the 3-year observation of students in High School B, the Kruskal–Wallis test showed a significant difference (*P* = 0.010; Table 1). The Bonferroni post hoc correction for multiple testing indicated that the 2013 SAS scores were significantly higher than the 2014 scores (*P* < 0.05). For the 2-year observation of High School C students, the Mann–Whitney-*U*-test showed no significant difference (*P* = 0.542; Table 1).

Regarding IES-R scores, for the 3-year observation of students in High School A, the Kruskal–Wallis test showed a significant difference (*P* = 0.000; Table 1). A Bonferroni post hoc correction for multiple testing indicated that the 2012 IES-R scores were significantly higher than those for 2014 (*P* < 0.05). For the 3-year observation of High School B students, the Kruskal–Wallis test showed a significant difference in IES-R scores (*P* = 0.000; Table 1). The Bonferroni post hoc correction for multiple testing indicated that the 2012 IES-R scores were significantly higher than the 2013 scores and the 2014 scores were significantly higher than the 2012 scores (*P* < 0.05). For the 2-year observation of High School C students, the Mann–Whitney-*U*-test showed that the 2012 IES-R scores were significantly higher than the 2013 scores (*P* = 0.000; Table 1).

### Longitudinal changes in physical behaviours

Regarding outside play time, in the 3-year observation of students in High Schools A and B, the Kruskal–Wallis test showed no significant difference (High School A *P* = 0.786; High School B *P* = 0.613; Table 2). In the 2-year observation of High School C students, the Mann–Whitney-*U*-test showed that outside play time had no significant difference between 2012 and 2013 (*P* = 0.662) (Table 2).

**Table 2:** Longitudinal changes in physical behaviours: outside play time, TV viewing time, and electronic gaming time.

Year of survey		High School A	(*n*)	High School B	(*n*)	High School C	(*n*)
*Outside play time*							
Total		1.00 [0–3]	1732	0.00 [0–1]	2240	0.50 [0–2]	1124
2012		1.00 [0–3]	596	0.00 [0–1]	707	0.25 [0–2]	477
2013		1.00 [0–3]	594	0.00 [0–1]	788	0.50 [0–2]	647
2014		1.00 [0–3]	542	0.00 [0–1]	745		
Kruskal–Wallis test	*χ*^2^	0.482		3.629			
	df	2		2			
	*P*	0.786		0.163			
Mann–Whitney test	U					152,103.50	
	*Z*					−0.437	
	*P*					0.662	
*TV viewing time*							
Total		5 [3–9]	1881	2 [1–3]	2363	2 [1–3]	1211
2012		2 [1–3]	641	2 [1–3]	749	2 [1–3]	524
2013		2 [1–3]	637	2 [1–3]	818	2 [1–3]^c^	687
2014		2 [1–3]	603	2 [1–2]^a^	796		
Kruskal–Wallis test	*χ*^2^	2.998		17.893			
	df	2		2			
	*P*	0.223		<0.001			
Mann–Whitney test	*U*					163,054.00	
	*Z*					−2.865	
	*P*					0.004	
*Electric gaming time*							
Total		0.67 [0–2]	1864	0.20 [0–1]	2292	0.00 [0–1]	1197
2012		0.50 [0–2]	635	0.00 [0–1]	740	0.00 [0–1]	509
2013		0.74 [0–2]	634	0.23 [0–1]	810	0.00 [0–1]	688
2014		1.00 [0–2]	595	0.50 [0–1]^a,b^	742		
Kruskal–Wallis test	*χ*^2^	5.950		23.333			
	df	2		2			
	*P*	0.051		<0.001			
Mann–Whitney test	*U*					174,343.00	
	Z					−0.139	
	*P*					0.889	

Numerical variables with skewed distributions are expressed as the median [25th–75th percentile].

^a^According to the post hoc Bonferroni test, there were differences in the scores between 2012 and 2014 (*P* < 0.05).

^b^According to the post hoc Bonferroni test, there were differences in the scores between 2013 and 2014 (*P* < 0.05).

^c^According to the post hoc Bonferroni test, there were differences in the scores between 2012 and 2013 (*P* < 0.05).

Regarding TV viewing time, in the 3-year observation of High School A students, the Kruskal–Wallis test showed no significant difference (*P* = 0.223; Table 2). In the 3-year observation of High School B students, the Kruskal–Wallis test showed a significant difference (*P* = 0.000; Table 2). The Bonferroni post hoc correction for multiple testing indicated that the 2012 TV viewing time was significantly longer than the 2014 time (*P* < 0.05). Finally, in the 2-year observation of High School C students, the Mann–Whitney-*U*-test showed that TV viewing time was significantly longer in 2012 than in 2013 (*P* = 0.004; Table 2).

Regarding electronic gaming time, in the 3-year observation of High School A students, the Kruskal–Wallis test showed no significant difference (*P* = 0.051; Table 2). In the 3-year observation of High School B students, the Kruskal–Wallis test showed a significant difference (*P* = 0.000; Table 2). The Bonferroni post hoc correction for multiple testing indicated that the 2014 electronic gaming time was significantly longer than the 2012 and 2013 times (*P* < 0.05). In the 2-year observation of High School C students, the Mann–Whitney-*U*-test showed that electronic gaming time was not significantly different between 2012 and 2013 (*P* = 0.889; Table 2).

Figure 4 depicts the differences in daily life activities due to tsunami damage. The time spent watching TV decreased significantly year-on-year in High Schools B and C, while the time spent playing electronic games increased significantly year-on-year in High Schools A and B.

### Three models of factors that contribute to resilience

We modelled the factors contributing to resilience by tsunami damage using Prediction One (Sony Communication Inc., Japan) (Fig. 5). In all tsunami damage models, the depressed mood score was the highest contributing factor to the resilience score.

## Discussion

The main contribution of this study, which is not common in the scientific literature, is the investigation of the psychological state and life characteristics of adolescents under stress, specifically students aged 15 to 18 years who were affected by the Great East Japan Earthquake and tsunami in the 3 years following the disaster. Our results may be applicable to improving the psychological state of adolescents under the current COVID-19 pandemic, thereby increasing their resilience.

Thus, interventions targeting depressed mood would be important to increase the resilience of adolescents under the COVID-19 pandemic. Counselling and medical interventions by child and adolescent psychiatrists could also be relevant in some cases. Avoiding sedentary lifestyles and incorporating exercise is recommended to improve depressed mood, and providing such information will be key in promoting psychological protection for adolescents during the pandemic. In some cases, these will need to be supplemented by the provision of information by schools; further, in some cases, regulations, environmental plans, and laws are warranted.

Outdoor play may be important to avoid a sedentary lifestyle. High School B and High School C, which suffered relatively little damage from the tsunami, contributed the third most to the resilience score, while High School A, which suffered the most damage from the tsunami, contributed the least to the resilience score. Students from High School A reduced outdoor play due to the aftermath of the tsunami. This indicates that outdoor play increases resilience thereby suggesting that COVID-19 infection control could affect adolescents’ resilience.

### Changes in psychological state in the 3 years following the Great East Japan Earthquake

**Fig. 3:**
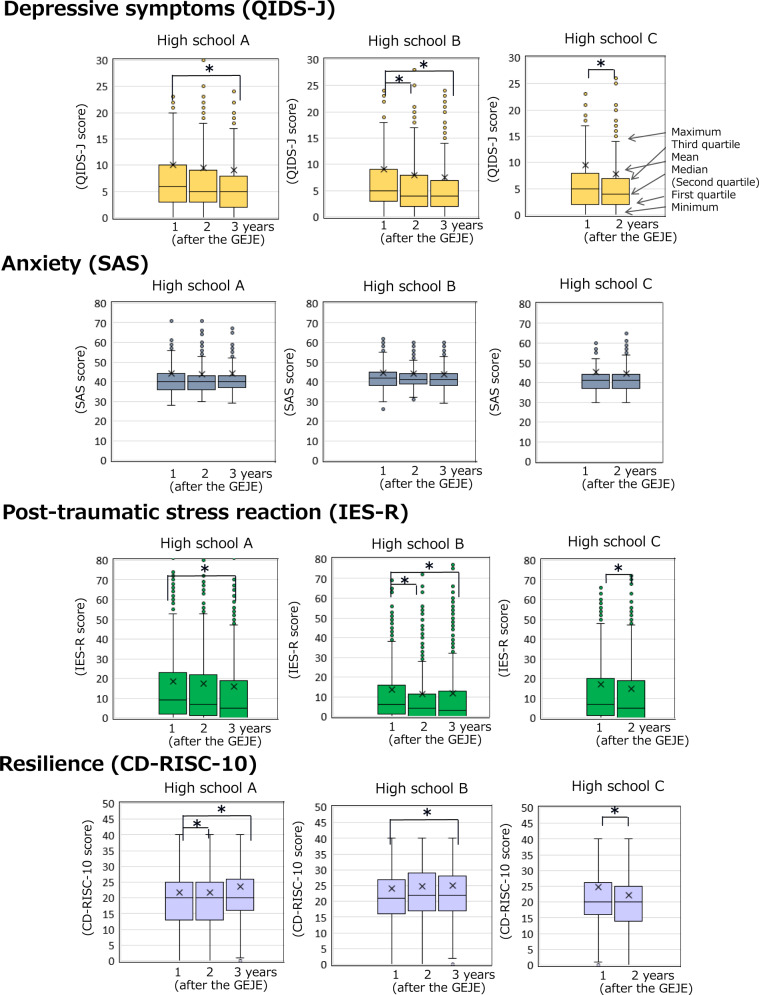
Resilience and mental health indicators of students assessed in 2012 to 2013. QIDS-J Quick Inventory of Depressive Symptomatology—Japanese version, SAS Zung Self-Rating Anxiety Scale, IES-R Impact of Event Scale—Revised, CD-RISC-10 10-item Conner-Davidson Resilience Scale, GEJE, Great East Japan Earthquake.

Resilience buffers the negative influences of stressful life events over time (Lyons and Parker, 2007). It was previously showed that annual changes in 2012–2014 and 2013–2014 resilience and depressive symptoms scores for students affected by the Great East Japan Earthquake were negatively correlated (Okuyama et al., 2018). The increase of CD-RISC-10 scores were related to the degree of damage caused by the Great East Japan Earthquake and the tsunami. Anxiety (SAS scores) did not change significantly in any high school from 2012 to 2014, but depressive symptoms (QIDS-J scores), post-traumatic stress reaction (IES-R scores), and resilience (CD-RISC-10 scores) changed significantly in all three high schools. Figure 3 indicates the psychological differences between 2012, 2013 and 2014 in the same high school.

As shown in Fig. 3, resilience scores decreased over time in High School C, that was unaffected by the tsunami. High School A, which was severely affected by the tsunami, reported a significant increase in resilience scores every year. High School B, which was moderately affected by the tsunami among the three schools, showed a significant increase in resilience in the third year after the disaster compared to the first year. This suggests that adolescents suffering traumatic symptoms from disasters such as the massive tsunami may recover over time by developing resilience. However, even after a major disaster, resilience may be lower among youth populations that are considered relatively less affected. This may be because there is less support for such youth due to less damage compared to other populations.

### Daily activities in the 3 years following the Great East Japan Earthquake

Social isolation due to COVID-19 caused a significant disruption in daily routines for the global community, especially adolescents. A Sicilian study reported that the lockdown negatively affected physical activity, with greater impacts on men and overweight persons (Giustino et al., 2020). A decrease in physical activity was expected because of social distancing (Gilic et al., 2020). It is also important for children and adolescents to participate in and enjoy physical activity during their leisure time (Shahidi et al., 2020).

**Fig. 4:**
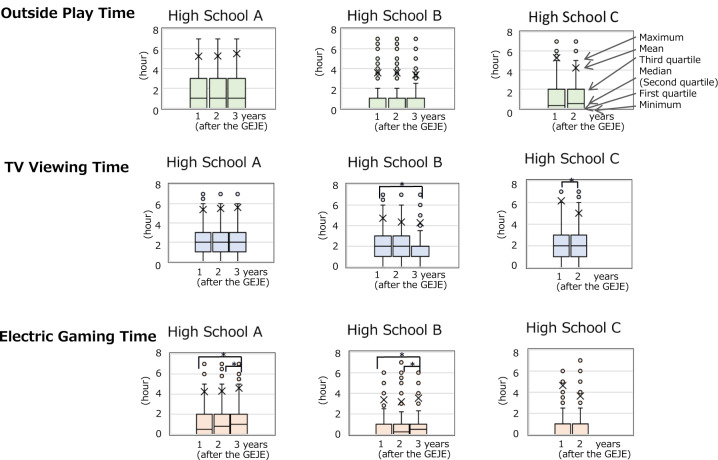
Differences in daily life activities due to tsunami damage. QIDS-J Quick Inventory of Depressive Symptomatology—Japanese version; SAS Zung Self-Rating Anxiety Scale, IES-R, Impact of Event Scale—Revised; CD-RISC-10, 10-item Conner-Davidson Resilience Scale; GEJE, Great East Japan Earthquake. *According to the Kruskal–Wallis test, there was a difference in time between 2012, 2013, and 2014 (*P* < 0.05); according to the Mann–Whitney *U*-test, there was a difference in time by between 2012 and 2013 (*P* < 0.05).

We present data on the lives of high-school students after the Great East Japan Earthquake. As shown in Fig. 4, differences in daily life activity were found according to tsunami damage: TV viewing time significantly decreased year-on-year in High Schools B and C, while electronic game time significantly increased year-on-year in High Schools A and B. Thus, after a major stressful event, young people may tend to lead sedentary lifestyles for extended periods.

### Applying post-earthquake data to the mental health of adolescents during the COVID-19 pandemic

As adolescents infected with COVID-19 generally reported lower disease severity than adult patients (Dong et al., 2020), Czeisler et al. (2020) conducted a survey of U.S. adults in June 2020 and found that the COVID-19 pandemic had a significant mental health impact on adolescents. Mental health of adolescents in China was examined two weeks after the COVID-19 outbreak and 40.4% were reported to have psychological problems (Liang et al., 2020; Cohen et al., 2021). Based on the results of this survey of high-school students after the Great East Japan Earthquake, it is expected that the psychological effects of the pandemic will continue for several years.

Several proposals have been made to alleviate the psychological problems of adolescents caused by the COVID-19 pandemic (Imran et al., 2020a; Imran et al., 2020b), but their effectiveness has not been tested. Conversely, interventions to improve the mental health of adolescents after natural disasters such as the Great East Japan Earthquake have already been evaluated in terms of the methods and their effectiveness (Okuyama et al., 2021b).

## Limitations and future directions

**Fig. 5:**
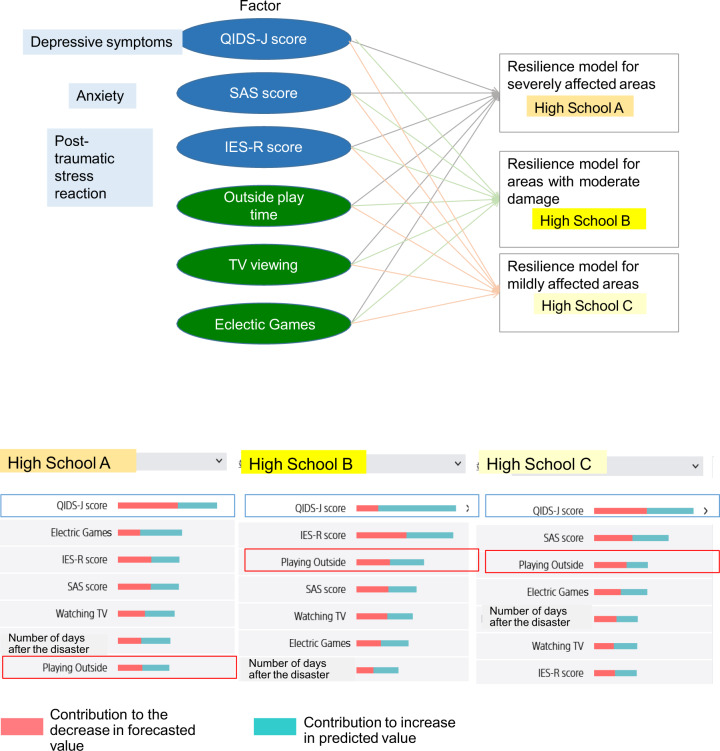
Factors contributing to resilience after the Great East Japan Earthquake. QIDS-J Quick Inventory of Depressive Symptomatology—Japanese version; SAS Zung Self-Rating Anxiety Scale, IES-R Impact of Event Scale— Revised, CD-RISC-10 10-item Conner-Davidson Resilience Scale.

This study focused on finding the factors involved in promoting mental health care after the Great East Japan Earthquake and increasing resilience as a result. Resilience is known as a protective factor against the onset of depression (Walker et al., 2022) and we explored factors that contributed to this resilience (Fig. 5). However, resilience might not solely be captured by the CD-RICK-10 we used. Although exact definitions vary by discipline, the American Psychological Association (2014) defines resilience as “the process of successfully adapting in the face of adversity, trauma, tragedy, threat, or significant sources of stress.” Originally, resilience was viewed as a stable personality characteristic that enhances the ability to adapt to adverse experiences (Luthar et al., 2000). Recently, however, resilience has been viewed as a dynamic process that varies throughout the life course (Davydov et al., 2010; Southwick, et al., 2015). Thus, since the construct itself is multifaceted in nature, there is a possible bias in assessing resilience only by CD-RISC-10 scores. Other concept-based measures of resilience may need to be used in conjunction.

This study used data obtained through a questionnaire survey, but future research should consider combining data obtained from interviews with children and adolescents under stress from the COVID-19 pandemic to fill in the gaps in the questionnaire-based study, enrich the data content, and obtain more comprehensive and detailed results.

Furthermore, high-school students in Japan are at a crossroads of their lives in terms of finding employment and entering university, making it difficult for them to devote the time they would normally spend on such a survey. Since this survey was conducted at a time when mental health care was the focus of attention after the Great East Japan Earthquake, as evidenced by an increase in the number of school counsellors at affected high schools, it is possible that cooperation of the three high schools was obtained, which made the survey collection rate high. Future surveys conducted in the same manner may have a lower response rate and not be indicative of the state of Japanese high-school students. Thus, in future studies, we will consider increasing the effectiveness of the obtained questionnaire results by adapting the model of the current study results. At the same time, we will also consider fitting different models, including various types of machine learning in our analysis. This will further enhance the research and allow for more reliable conclusions.

## Data Availability

The datasets generated and/or analysed during the current study are not publicly available due to privacy and ethical restrictions but are available from the corresponding author on reasonable request.
